# EEFSA-SECM: an enhanced ensemble feature selection and stacking ensemble classifier to detect Parkinson’s disease

**DOI:** 10.3389/fneur.2026.1717252

**Published:** 2026-03-02

**Authors:** Vridhi Rajput, N. Maheswari

**Affiliations:** School of Computer Science and Engineering, Vellore Institute of Technology, Chennai, India

**Keywords:** classifier, ensemble, feature selection, Parkinson’s disease, speech

## Abstract

**Introduction:**

Parkinson’s disease (PD) is a progressive neurological disorder whose early symptoms often remain undetected, making timely diagnosis challenging. Machine learning offers strong algorithms to detect subtle speech-based biomarkers that are impossible to detect by standard methods.

**Methods:**

In this article, we proposed an Enhanced Ensemble Feature Selection Algorithm (EEFSA) which combines filter, wrapper, and embedded approaches to extract the best informative features, eliminate redundancy, and improve classification performance. The proposed work has tested on two benchmark audio based datasets, such as Dataset-1 (46 features, 80 samples), where EEFSA reduced the features to 20 features, and Dataset-2 (754 features, 252 samples), where EEFSA reduced the features to 40 features. Nine machine learning classifiers were tried out and the best of them were combined into a stacking ensemble with logistic regression as the meta-classifier.

**Results:**

Experiments show that EEFSA-driven dimensionality reduction not only enhanced accuracy of classification but also reduced training time considerably and minimized over fitting. The Stacking Ensemble Classifier Model (SECM) deployed on the basis of the proposed method achieved accuracy of 86.67% and 89.95% on Dataset-1 and Dataset-2, respectively, and outperformed individual classifiers in all experiments.

**Conclusion:**

Overall, this work provides EEFSA-driven stacking as a new and efficient method of feature selection and ensemble learning combination for Parkinson’s disease classification. The proposed EEFSA–SECM framework achieves effective classification accuracy, competitive training/testing times, and improved AUC scores on two benchmark datasets, establishing it as an effective and efficient approach for Parkinson’s disease diagnosis.

## 1 Introduction

Parkinson’s disease (PD) is the second most frequent worldwide neurodegenerative disorder, present in about 2%–3% of individuals older than 65 and more than 8.5 million individuals worldwide (1). PD is diagnosed by insidiously progressive motor signs of tremor, rigidity, bradykinesia, and postural instability and a wide range of non-motor signs ranging from cognitive impairment, depression, sleep disorder, to fatigue (2). The underlying pathology is strongly associated with dopaminergic neuronal loss in the substantia nigra and abnormal *α*-synuclein aggregation, although the exact etiology remains a complex interplay of genetic and environmental factors. As incidence is projected to double in 2040, PD is fast becoming a worldwide health burden.

Early detection of PD is important as treatment like drug and rehabilitative therapies are more effective when not severe, administered at the early stages of the disease (2, 3). Diagnosis is still challenging, though, given that early symptoms of PD are similar to normal aging and other neurologic diseases (4). Conventional diagnostic approaches—largely dependent on clinical evaluation, patient history, and neuroimaging—are generally subjective, expensive, and too insensitive for the early detection of PD (4). This limitation in diagnosis has generated growing interest in leveraging non-invasive biomarkers, i.e., speech, handwriting, and gait patterns, to create computer-based diagnosis systems (3). Most importantly, voice analysis has been found to be an extremely promising modality, as nearly 90% of PD patients develop voice impairments like dysphonia, hypophonia, and speech mono tonic speech (5).

Machine Learning (ML) and Deep Learning (DL) techniques have proven effective for PD detection by capturing complex patterns in biomedical signals that are often beyond human observation (6). Voice-based datasets analysis has been found to exhibit greater than 95% accuracy, while enhanced performance has been seen through newer techniques such as feed-forward neural and kernel-based SVMs (1, 4). Furthermore, new studies emphasize the increasing importance of multimodal methods that combine speech, locomotion, and imaging in order to produce predictions that are more reliable and accurate (7).

Among the ML techniques, ensemble learning has also been demonstrated to enhance diagnostic performance strongly. Techniques such as bagging, boosting, and stacking leverage the merit of an ensemble of classifiers in reducing variance, bias, and overfitting (8, 9). Stacking, indeed, was demonstrated to be effective for PD prediction tasks by combining a set of diverse base learners (such as Random Forest, XGBoost, and neural networks) to form a meta-classifier (5). For instance, Balaha et al. (7) attained almost perfect accuracies utilizing stacking ensembles on multimodal datasets, while the authors (5) demonstrated the improved robustness of classifiers by using stacking in voice-based PD detection.

### 1.1 Contributions

- An Enhanced Ensemble Feature Selection Algorithm (EEFSA) is proposed and it integrates filter, wrapper, and embedded methods to select highly discriminative and non-redundant features from Parkinson’s disease voice datasets.
- The effectiveness of EEFSA is evaluated by comparing the performance of nine machine learning classifiers on both full and reduced feature sets, demonstrating consistent improvements in accuracy, generalization, and computational efficiency.
- To address class imbalance, the SMOTE technique is applied, ensuring fair representation of minority classes and enhancing classifier robustness.
- A Stacking Ensemble Classifier Model (SECM) is constructed using the top-performing base learners with Logistic Regression as a meta-classifier, significantly outperforming individual classifiers.
- The proposed EEFSA–SECM framework achieves superior classification accuracy, competitive training/testing times, and improved AUC scores on two benchmark datasets, establishing it as an effective and efficient approach for Parkinson’s disease diagnosis.

## 2 Literature review

The precise diagnosis of Parkinson’s disease (PD) is one of the emerging machine learning applied research domains due to the automation of PD classification. In order to improve diagnostic accuracy, the most recent studies propose the use of ensemble methods, along with advanced feature selection techniques incorporating multimodal data with the primary PD. This review outlines approaches for PD classification emphasizing the use of ensemble learning, feature selection methods, and the examination of relevant computation.

Recent studies suggest a significant enhancement in PD classification using ensemble techniques. Velmurugan and Dhinakaran (5) used a stacked ensemble model with base classifiers such as Decision Trees, Random Forests, and SVMs and a meta classifier to combine the outputs for predicting classification of PDs. Their findings showcased that, stacking offered greater accuracy and stability than the individual classifiers highlighting the importance of ensemble learning techniques to develop reliable and accurate automated systems for medical diagnosis. Shastry (9) proposed an ensemble of k-Nearest Neighbors (KNN) and Gradient Boosting (GB) called Nearest Neighbor Boosting (NNB) for PD detection. NNB with F-PER, a Feature Permutation technique outperformed the standard classifiers in both accuracy and confidence. This further strengthens the claim for hybrid ensemble models that integrate distance-based and boosting techniques.

Ghaheri et al. (10) proposed a PD detection model using voice datasets, They performed feature selection using SHAP along with a hard voting ensemble classifier. SHAP selected important features and then based on the output of individual classifiers ensemble classifier was built. In comparison to the individual models, their model showed better performance. Therefore, the integration of feature selection and ensemble methods provide more accuracy and reliability. Ali et al. (11) proposed EOFSC, an Ensemble of Optimal Features with Sample-dependent Classifiers. This model examined specific types of phonation in voice recordings of PD patients and computed optimal feature subsets for each phonation type. Their model, using tailored classifiers for these subsets, outperformed models using single classifiers by 6.5%. This highlights the significance of using diverse phonation data for accurate PD detection.

There has been recent focus on the role of feature selection methods toward improving the performance of PD classification systems. Nahar et al. (12) applied ensemble bagging classifiers along with wrapper feature selection for detecting Parkinson’s disease. Nahar et al. (12) showed that feature selection enhances classification accuracy. The classifiers trained on the lower-dimensional datasets were achieved 82% accuracy, confirming the hypothesis that detection performance improves with the use of relevant feature subsets. However, their study was solely focused on bagging-based ensemble methods and did not explore other more sophisticated ensemble approaches.

In a more comprehensive study, Qasim et al. (13) developed a hybrid framework for PD based on Recursive Feature Elimination (RFE), Principal Component Analysis (PCA) and SMOTE combined. They evaluated multiple classifiers on the UCI PD voice dataset and SVM outperformed all other classifiers, with an accuracy of 98.2%. The study demonstrated that robust classifiers could be constructed with aggressive resampling and reduction in dimensions, however, but the complex hybrid structure still poses an excessive computational burden.

Sabeena et al. (14) suggested an Optimization Based Ensemble Feature Selection Algorithm with a hybrid search method to extract voice features for Parkinson’s disease (PD) classification using deep neural networks. Their approach provided better precision, recall, and feature selection performance, with reduced overfitting tendencies. The approach suffered from higher computation complexity, reiterating the classic compromise between performance and efficiency.

Deep learning methods along with traditional machine learning methods have provided effective results for detection of PD. Wang et al. (15) employed this approach and applied it on multimodal movement and speech signals for early detection of PD. They showcased deep learning’s potential for PD detection with a 96% accuracy, achieved with SVM, RF, and deep neural networks. However, there were some limitations such as a lack of sufficient computing resources, which affected practical implementation.

Al-Sarem et al. (16) used CatBoost, a gradient boosting algorithm, on Parkinson’s disease voice data and used feature importance scoring to select the most discriminative acoustic parameters. Their model performed competitively relative to the results produced by Random Forest and Logistic Regression. This work proves the importance of advanced boosting techniques and feature selection for PD diagnosis and underscores the importance of features in efficiently narrowing datasets to vital features.

This research work explores an enhanced stacking-based ensemble approach combined with an Ensemble Feature Selection Algorithm (EEFSA) to diagnose the Parkinson’s disease in the early stage. It addresses the challenges of small dataset size, class imbalance, and feature redundancy issues—providing a more powerful, non-invasive, and scalable Parkinson’s disease diagnosis solution.

## 3 Methodology

In this study, a framework has proposed to detect the Parkinson’s disease (PD) in the early stage using audio data. The data for this study was collected from the UCI Machine Learning Repository. The framework consists of two phases: the first phase involves applying machine learning classifiers with no feature reduction, and the second phase utilizes an Enhanced Ensemble Feature Selection Algorithm (EEFSA) that combines several techniques—filter, wrapper, and embedded methods to feature selection. Biomedical datasets, when modeled with machine learning algorithms, often suffer from the curse of dimensionality, introducing redundancy and decreasing the model’s efficacy.

To solve the class imbalance problem, we used the SMOTE algorithm to construct balanced datasets. After achieving a balanced dataset, optimized classifiers were used to classify the PD and healthy subjects. The proposed system has a Stacking Ensemble Classifier Model (SECM), that has multiple base learners, such as Random Forest, XGBoost, Support Vector Machines, and Multilayer Perceptron. The results of the base learners are aggregated by a meta classifier to improve the robustness.

**Figure 1:**
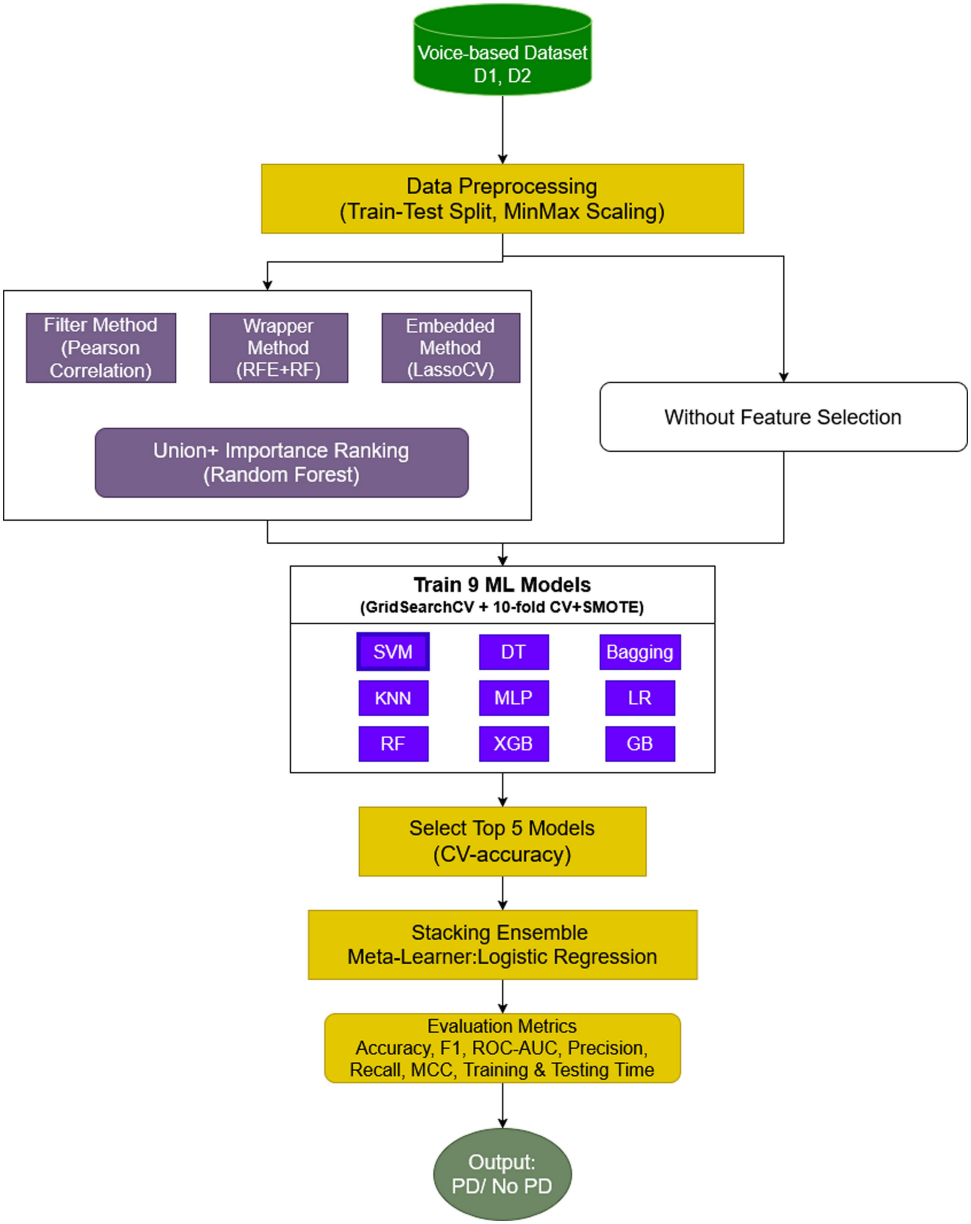
Proposed framework.

Later, a detailed evaluation of the models supports an extensive analysis using accuracy, precision, recall, F1-score, and AUC. The overall workflow of the proposed work is illustrated (Figure 1) and it shows the integration of EEFSA, SMOTE, and stacking ensemble learning to achieve the accurate and efficient audio based PD prediction.

### 3.1 Dataset

This proposed work demonstrates the functioning capabilities of the Enhanced Ensemble Feature Selection and Aggregation (EEFSA) framework, that takes two distinct datasets of Parkinson’s disease. This multiple dataset method is helpful to strengthen the analysis on varying demographic and acoustic data profiles.

Dataset 1: The dataset has collected from the UCI Machine Learning Repository (17), which has 240 voice based recording instances of 80 persons, 40 with Parkinson’s Disease (PD) and 40 with healthy persons. The healthy persons group in the dataset has 22 males and 18 females with a average age of 66.38, and the PD persons group has 27 males and 13 females with a average age of 69.58. This dataset contains 46 acoustic features which can be categorized into five groups: pitch perturbation, amplitude perturbation, spectral envelope measures, noise features, and nonlinear measures. Important features such as jitter and shimmer (weighted averaging) are critical for PD symptom (resting tremor, bradykinesia, and rigidity) detection and are measured using sophisticated voice signal processing algorithms.

Dataset 2: The second dataset used in this research was collected from the voice recordings of patients in the Department of Neurology, Cerrahpasa Faculty of Medicine, Istanbul University (18, 28). The dataset consists of voice recordings of 252 people which include 188 patients diagnosed with Parkinson’s disease (107 males and 81 females, aged 33–87; mean age: 65.1 ± 10.9) and 64 healthy control subjects (23 males and 41 females, aged 41–82; mean age: 61.1 ± 8.9). Participants were clinically examined and were instructed to perform sustained phonation of the vowel a three times and were recorded at a 44.1 kHz sampling rate.

This dataset contains 756 instances and 754 features, with the features consisting of both integer and real-valued numbers, with no missing values. In order to derive pertinent acoustic features for the clinical evaluation of Parkinson’s disease, speech signal processing methods were utilized such as Time-Frequency Features, Mel-frequency cepstral coefficients (MFCCs), wavelet transform-based features, vocal fold features, and Teager Wavelet Quantization Transform features (TWQT). These features highlight PD-related vocal changes, including the PD symptoms of vocal tremor, rigidity, and bradykinesia, which enhances the dataset’s utility for classification tasks.

### 3.2 Data preprocessing

In the context of the frameworks of machine learning, the need for data pre-processing is crucial with regard to trust and effectiveness. For this analysis, various pre-processing steps were carried out on the voice dataset of patients with Parkinson’s disease. To eliminate any irrelevant or missing information appropriate cleaning was performed.

To maintain class balance and the distribution of different categories for training and testing in the model, the dataset was split using a subject-independent stratified group split sampling resulting in 75% for training and 25% for testing. The MinMaxScaler was used for feature scaling, which scales up for every feature to lie between the range of 0–1. The scaler was fitted exclusively on the training data and subsequently applied to the test data using the same scaling parameters.

Considering the dataset’s medical context and possible class imbalance, the training dataset was improved using SMOTE (Synthetic Minority Over-sampling Technique). SMOTE was applied only within the training portion of each cross-validation fold, using default configuration (*k* = 5 nearest neighbors) and a fixed random seed (random_state = 42) ensuring that no synthetic samples were generated from validation or test data. This approach helps deal with the imbalance during training, which enhances the model’s robustness and increases generalization performance, reducing bias in scenarios where the classifier does not handle imbalance well. Despite the global class balance of Dataset-I, subject-independent cross-validation can induce fold-level imbalance arising from subject-level grouping and unequal sample distributions across folds.

### 3.3 Enhanced ensemble feature selection algorithm (EEFSA)

**Figure 2:**
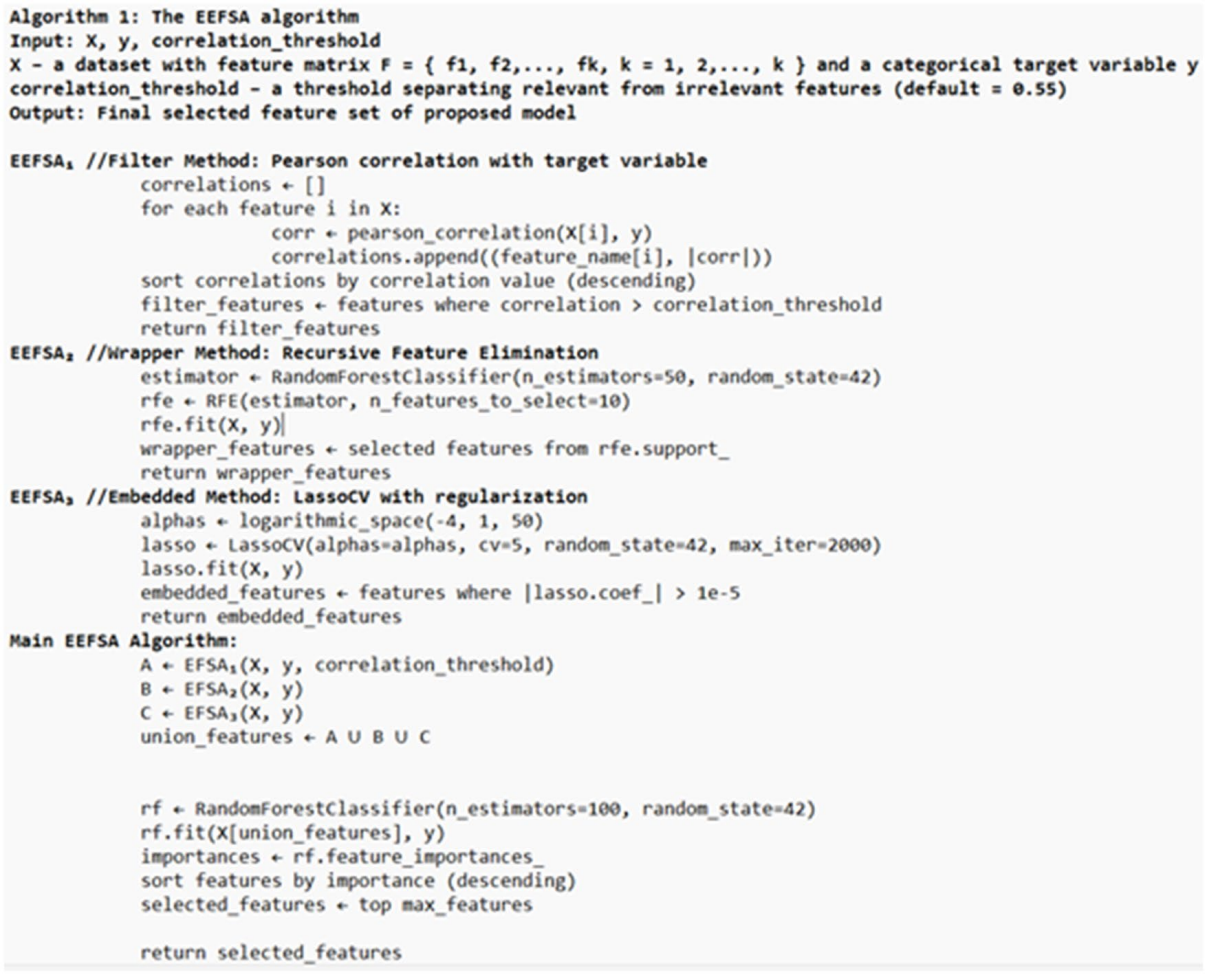
Enhanced ensemble feature selection algorithm (EEFSA).

**Figure 3:**
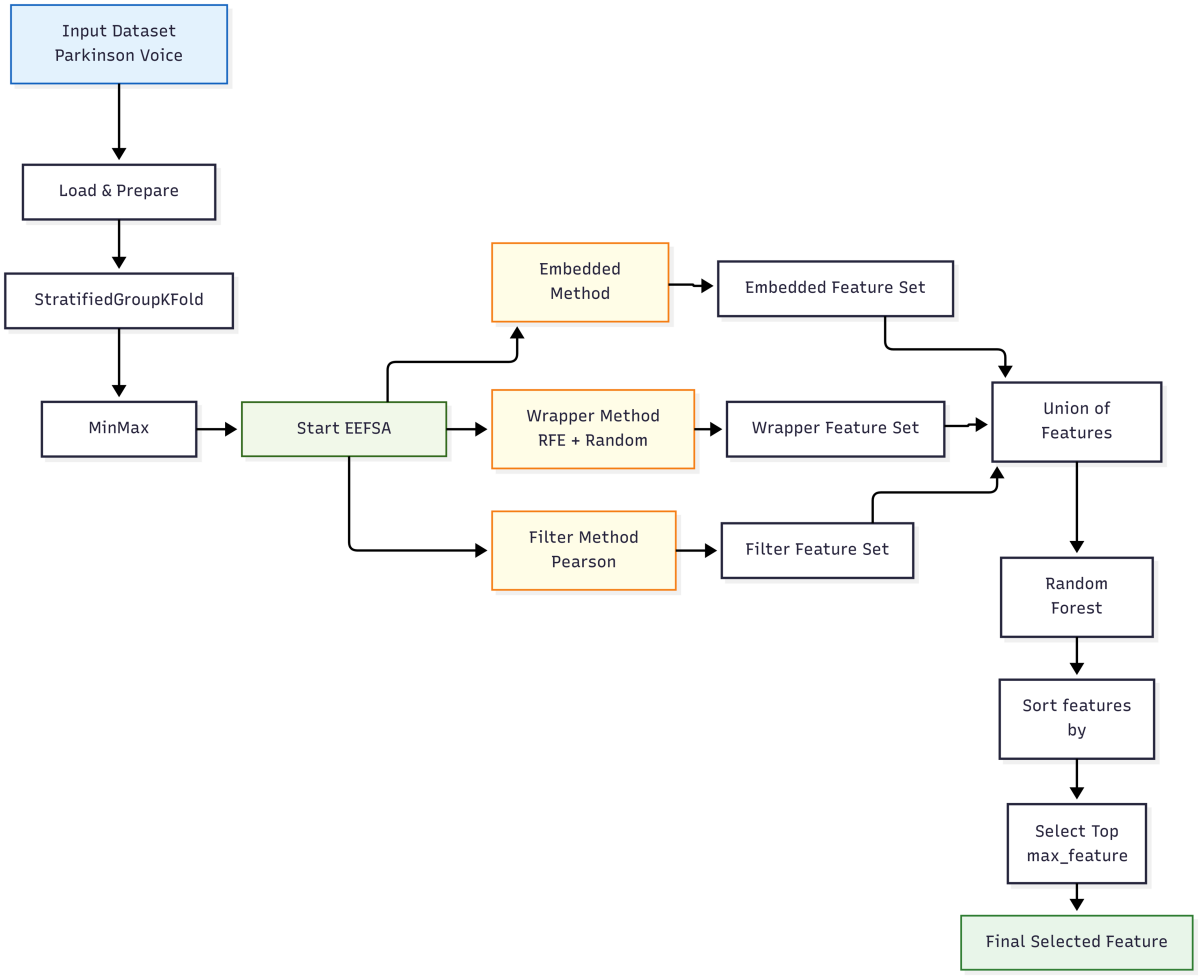
Enhanced ensemble feature selection.

In this research, we implemented a custom Ensemble Feature Selection Algorithm, based on Singh and Tripathi (19), to improve the classification of Parkinson’s disease using voice data. Unlike, the original EEFSA that used backward elimination as a wrapper, we used RFE with the Random Forest estimator as a wrapper and to obtain a more structured and stable wrapper-based feature selection process. Backward elimination removes the features sequentially based on the statistical significance from the regression model and it does not consider the features later, when removed. This lead to the selection of suboptimal feature subsets (20). RFE is a structured type of wrapper method that repeatedly retrains the model and updates the feature rankings at each elimination step, that leads to the suitable and reliable feature selection (21). When combining with RFE, it takes the tree-based importance measures that can capture nonlinear relationships and feature interactions, that results in stable feature rankings (22). EEFSA as mentioned in Algorithm 1 (Figures 2, 3), integrates three diverse selection techniques—Pearson Correlation (filter), RFE (wrapper), and LassoCV (embedded)—to identify influential features. The union of selected subsets was further refined using Random Forest-based importance ranking to retain the top features. This ensemble method resulted in elimination of irrelevant and redundant labels, increasing the accuracy of the classification. The refined EEFSA strategy not only improves classification accuracy and reduces overfitting but also enhances the model’s ability to generalize across folds.

### 3.4 Stacking ensemble classifier model (SECM)

**Figure 4:**
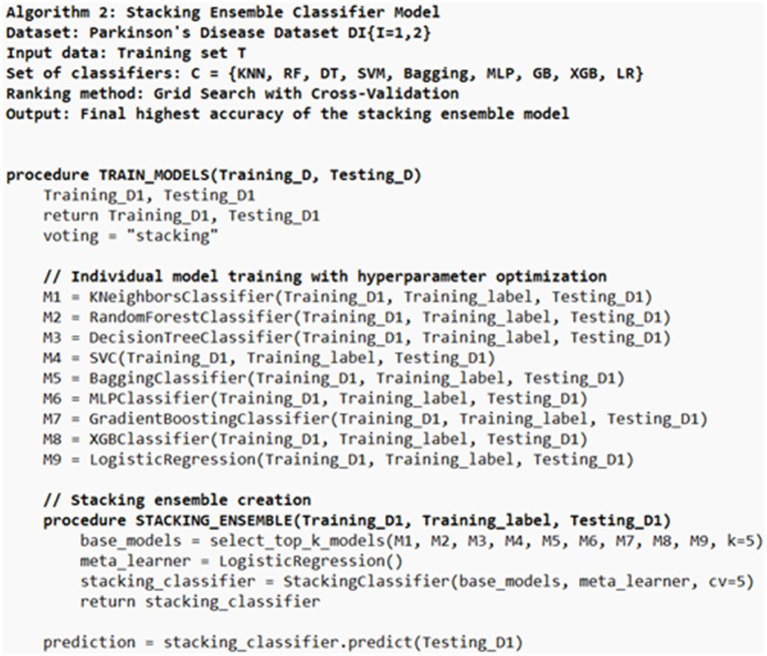
Stacking ensemble classifier model (SECM).

**Figure 5:**
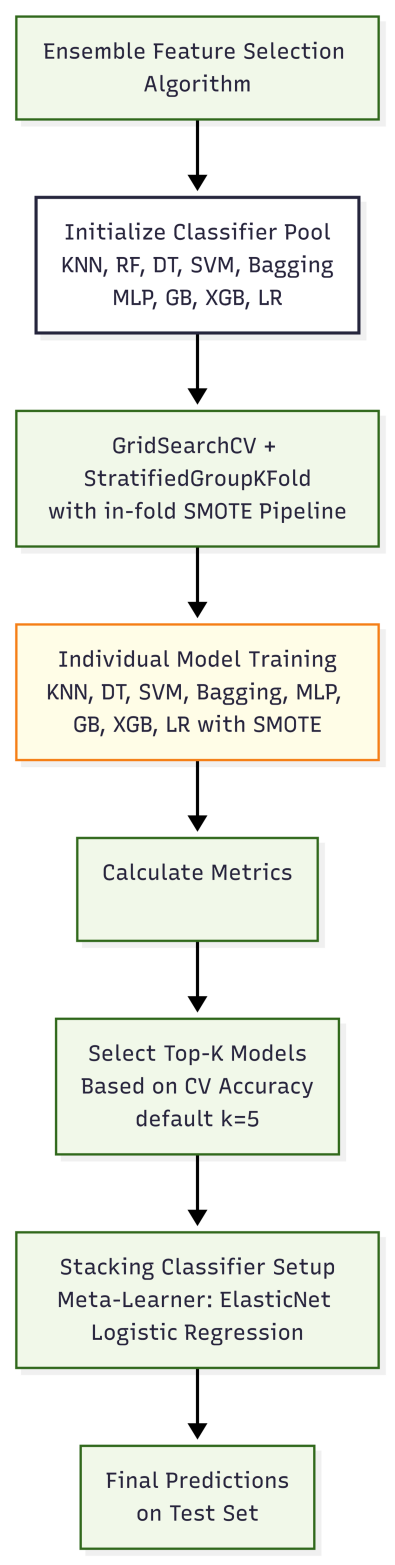
Stacking ensemble classifier.

For the classification of Parkinson’s disease, this research used a set of nine well-known machine learning classifiers: K-Nearest Neighbours, Random Forest, Decision Tree, Support Vector Machine, Bagging (Decision Trees as base estimators), Multi-Layer Perceptron, Gradient Boosting, XGBoost, and Logistic Regression. Each model was thoroughly hyper parameter tuned using GridSearchCV with 10-fold cross-validation to determine the best parameter settings. Models with the best cross-validated accuracy scores were five and form a stacking ensemble classifier as in Algorithm 2 (Figures 4, 5). A logistic regression model was then used to train the final meta-classifier on the ensemble’s base learners predicted probabilities. Thus, the ensemble approach was constructed.

#### 3.4.1 Hyperparameters of ML models

Within this study, each machine learning classifier used required one or more hyperparameters to manage their learning processes and, in turn, affect prediction accuracy. As with every machine learning model, finding the optimal combination of hyperparameter values is crucial since poor choices may result in the model either failing to accurately capture the patterns in the data or becoming too complex. In this study, GridSearchCV with 10-fold cross-validation was employed to automatically find optimal settings for all model hyperparameters, which guarantees reliable and unbiased model evaluation while the models are being trained.

After hyperparameter tuning, every single classifier was ranked according to their cross-validation accuracy. The top five models were selected and placed into a stacking ensemble. In SECM, the base learners’ outputs are combined using class probability predictions. Each base classifier produces predicted class probabilities, which are then used as input features for training the meta-classifier.

For Dataset-I, Logistic Regression is used as a meta-learner and the base learners’ prediction probabilities are used as inputs configured with maximum of 2000 iterations and a fixed random seed for reproducibility. Dataset-II used Logistic Regression with elastic-net regularization, configured with SAGA solver, a regularization strength of C = 5.0, an L1 ratio of 0.5, a maximum of 3,000 iterations. This configuration enables effective regularization and stable convergence when aggregating probability outputs from heterogeneous base classifiers.

This ensemble is aimed at improving accuracy by taking full advantage of the strengths and minimizing the weaknesses of the classifiers.

### 3.5 Performance evaluation

The Ensemble and the rest of the classifiers were trained on Parkinson datasets with the voice features extracted and evaluated using conventional classification techniques. Accuracy as in Equation 2 is one of the most basic K evaluation measurements. Precision as in Equation 1 is the ratio of correctly detected positive samples to all samples, the Recall (TPR) is the ratio of positives to actual positives, which tells us what proportion of real Parkinson’s cases were captured. The F1 score as in Equation 4 which is defined as the average of the precision and recall as in Equation 3 in ratio’s proportion adds a balanced dimension to the evaluation especially when there exists a class imbalance phenomenon.

The True Negative Rate (TNR) also analyses the classifier’s capability of identifying the healthy controls; the classifier’s misclassification tendencies are described by the False Positive Rate (FPR) and False Negative Rate (FNR). To calculate the performance based on all four components of confusion matrix, the Matthews Correlation Coefficient (MCC) as in Equation 5 can be used, making it very relevant for the datasets that are not evenly distributed. The summary of the discriminative power of the models at all thresholds is given by The Area Under the Receiver Operating Characteristic Curve (AUC-ROC) as in Equation 6. The higher the value the better the separation between the classes for the models. In assessing model stability, the 10-fold cross-validation accuracy was calculated, while the training and testing time was measured for assessing the computational efficiency.

The evaluation metrics are formally defined as follows:


(1) $$\text{Precision}=\frac{\mathrm{TP}}{\mathrm{TP}+\mathrm{FP}}$$

(2) $$\text{Accuracy}=\frac{\mathrm{TP}+\mathrm{TN}}{\mathrm{TP}+\mathrm{TN}+\mathrm{FP}+\mathrm{FN}}$$

(3) $$\text{Recall}(\mathrm{TPR})=\frac{\mathrm{TP}}{\mathrm{TP}+\mathrm{FN}}$$

(4) $$F1-\text{score}=\frac{2\times \text{Precision}\times \text{Recall}}{\text{Precision}+\text{Recall}}$$

(5) $$\mathrm{MCC}=\frac{\left(\mathrm{TP}\times \mathrm{TN})-(\mathrm{FP}\times \mathrm{FN}\right)}{\sqrt{\left(\mathrm{TP}+\mathrm{FP})(\mathrm{TP}+\mathrm{FN})(\mathrm{TN}+\mathrm{FP})(\mathrm{TN}+\mathrm{FN}\right)}}$$

(6) $$\mathrm{AUC}-\mathrm{ROC}=\int_{0}^{1}\mathrm{TPR}(\mathrm{FPR})d(\mathrm{FPR})$$

where TP, TN, FP, and FN denote true positives, true negatives, false positives, and false negatives, respectively.

These metrics jointly ensure a balanced evaluation of models in terms of classification accuracy, robustness, generalization, and computational efficiency.

## 4 Experimental results

### 4.1 Experiment—Dataset-I

This section analyses the evaluation of classifiers trained on Dataset I, considering Enhanced Ensemble Feature Selection and Aggregation (EEFSA) on model performance. Three feature selection algorithms were implemented based on filter, wrapper, and embedded methods, specifically Pearson Correlation, Recursive Feature Elimination (RFE), and LassoCV. A Stacking Ensemble Classifier (SECM) was created and combined with other machine learning classifiers to classify patients with Parkinson’s disease and healthy controls. Nine classifiers were trained which included the stacking ensemble with hyperparameter optimization using GridSearchCV method. A cross-validation procedure with 10 folds was performed to validate the results, and the average cross-validation score and its standard deviation were calculated. The evaluation metrics included accuracy, recall, F1 score, precision, ROC-AUC, and validation scores.

Initially, features with a correlation coefficient greater than 0.55 as mentioned in the Algorithm 1 were filtered using Pearson Correlation, resulting in 7 relevant features being selected. This correlation analysis was performed only on the training data to avoid information leakage. Then, Recursive Feature Elimination (RFE) with a Random Forest estimator was applied configured with 50 decision trees (*n*_estimators = 50) and a fixed random seed, and 10 influential features were selected, which applies the wrapper method. As the third method, embedded method LassoCV was used to compute the best regularization parameter using an alpha grid ranging from 10^−4^ to 10^1^, with 5-fold cross-validation, a maximum of 4,000 iterations, and a fixed random seed to select the 10 relevant features.

The union of these three subsets was ranked using Random Forest impurity-based feature importance scores, using Random Forest as the base estimator with 100 decision trees (*n*_estimators = 100) and the 20 most important features were selected to create the EEFSA subset for more efficient and accurate model training. The Stacking Ensemble Classifier Model (SECM) achieved greater accuracy scores using ensemble-selected features. The EEFSA method that selected 20 features outperformed the competing individual feature selection methods.

The EEFSA-selected features for Dataset-I highlight speech characteristics that are known to be affected in Parkinson’s disease. Perturbation features such as Jitter_PPQ and Shimmer_APQ5 capture instability in pitch and loudness, which are common in PD due to impaired vocal fold control. Noise-related measures including GNE, HNR35, and HNR38 reflect breathy and weak voices, often seen in patients with hypophonia.

Nonlinear features like RPDE, DFA, and PPE measure irregular and reduced pitch variation which helps capture unstable and monotone speech. In addition, several MFCC and delta MFCC features represent spectral and temporal changes in speech that are linked to articulatory imprecision and reduced speech clarity. All of these features describe various aspects of speech in the advanced state of Parkinson’s, explaining the improved classification performance achieved using EEFSA. The complete list of features selected at each stage of the EEFSA process for Dataset-I is provided in the Supplementary material. Classifiers were evaluated based on the full feature set and the EEFSA-reduced subset. The stacking ensemble was optimized with GridSearchCV for hyperparameter tuning, ensuring all classifiers were evaluated under the same experimental conditions. For Dataset-I, the final stacking ensemble comprised the top five classifiers selected based on cross-validation accuracy, namely SVM, Logistic Regression, Random Forest, KNN, and Bagging, as determined during model training. The optimized ensemble was implemented for all classifiers using the EEFSA-reduced set. The comparative performance is provided in Table 1, revealing how all classifiers have been trained on the full feature set opposed to the EEFSA-selected subset.

**Table 1:** Performance comparison of classifiers on Dataset-I without EEFSA feature selection.

Model	Accuracy (%)	F1-score (%)	Precision (%)	Recall (%)	MCC (%)	AUC	CV accuracy (10-fold) %	CV AUC (10-fold) %	Training time (sec.)	Test time (sec.)
KNN	85.00	84.75	86.21	83.33	70.04	0.941	75.00 ± 11.18	83.52 ± 11.35	7.74	0.0132
Random forest	81.67	81.97	80.65	83.33	63.37	0.919	78.89 ± 11.60	84.44 ± 10.33	38.11	0.0194
Decision tree	66.67	64.29	69.23	60.00	33.63	0.754	70.56 ± 14.07	72.47 ± 14.01	2.06	0.0037
SVM	86.67	86.67	86.67	86.67	73.33	0.944	80.00 ± 12.96	86.05 ± 11.10	3.94	0.0351
Bagging	81.67	81.36	82.76	80.00	63.37	0.894	79.44 ± 12.92	85.25 ± 11.87	50.67	0.0676
MLP	66.67	65.52	67.86	63.33	33.41	0.758	70.56 ± 10.26	78.89 ± 13.92	65.34	0.0056
Gradient boosting	81.67	81.36	82.76	80.00	63.37	0.866	75.00 ± 9.38	81.11 ± 10.85	97.01	0.0050
XGBoost	76.67	76.67	76.67	76.67	53.33	0.840	74.44 ± 10.89	82.10 ± 9.82	38.47	0.0382
Logistic regression	85.00	85.71	81.82	90.00	70.35	0.947	80.00 ± 11.97	84.94 ± 11.77	10.06	0.0036
Stacking ensemble	85.00	85.25	83.87	86.67	70.04	0.946	79.44 ± 10.26	85.06 ± 10.19	5.90	0.1674

Without feature selection, Support Vector Machine (SVM) achieved the highest test accuracy of 86.67%, followed by Logistic Regression and KNN with 85.00% accuracy, while the stacking ensemble also achieved an accuracy of 85.00% (Table 1). In terms of discriminative performance on the held-out test set, the stacking ensemble demonstrated competitive performance with a ROC-AUC of 0.946, which is comparable to SVM (0.944) and Logistic Regression (0.947).

After applying EEFSA, the stacking ensemble achieved the best overall test performance, obtaining an accuracy of 86.67%, F1-score of 87.10%, precision of 84.38%, recall of 90.00%, and the highest MCC of 73.50 among all classifiers (Table 2). In addition, the stacking ensemble maintained strong discriminative ability with a ROC-AUC of 0.943.

**Table 2:** Performance comparison of classifiers on Dataset-I with EEFSA feature selection.

Model	Accuracy (%)	F1-score (%)	Precision (%)	Recall (%)	MCC (%)	AUC	CV accuracy (10-fold)	CV AUC (10-fold)	Training time (sec.)	Test time (sec.)
KNN	81.67	82.54	78.79	86.67	63.65	0.853	78.89 ± 10.77	80.83 ± 11.52	6.21	0.0476
Random forest	76.67	77.42	75.00	80.00	53.45	0.910	79.44 ± 11.11	85.31 ± 11.71	40.25	0.0326
Decision tree	60.00	61.29	59.38	63.33	20.04	0.623	68.89 ± 12.22	70.68 ± 11.21	1.48	0.0037
SVM	83.33	83.87	81.25	86.67	66.82	0.940	82.78 ± 10.08	87.04 ± 9.21	2.23	0.0064
Bagging	75.00	74.58	75.86	73.33	50.03	0.858	78.33 ± 10.67	84.63 ± 11.01	35.49	0.0467
MLP	76.67	74.07	83.33	66.67	54.43	0.857	72.22 ± 8.99	84.20 ± 14.41	80.34	0.0042
Gradient boosting	71.67	71.19	72.41	70.00	43.36	0.832	76.67 ± 10.18	83.21 ± 9.61	61.16	0.0180
XGBoost	73.33	72.41	75.00	70.00	46.77	0.854	76.11 ± 13.16	83.70 ± 12.23	23.29	0.0121
Logistic regression	81.67	82.54	78.79	86.67	63.65	0.932	81.67 ± 10.26	86.42 ± 9.94	3.11	0.0051
Stacking ensemble	86.67	87.10	84.38	90.00	73.50	0.943	81.11 ± 9.36	86.30 ± 9.08	6.38	0.1318

**Figure 6:**
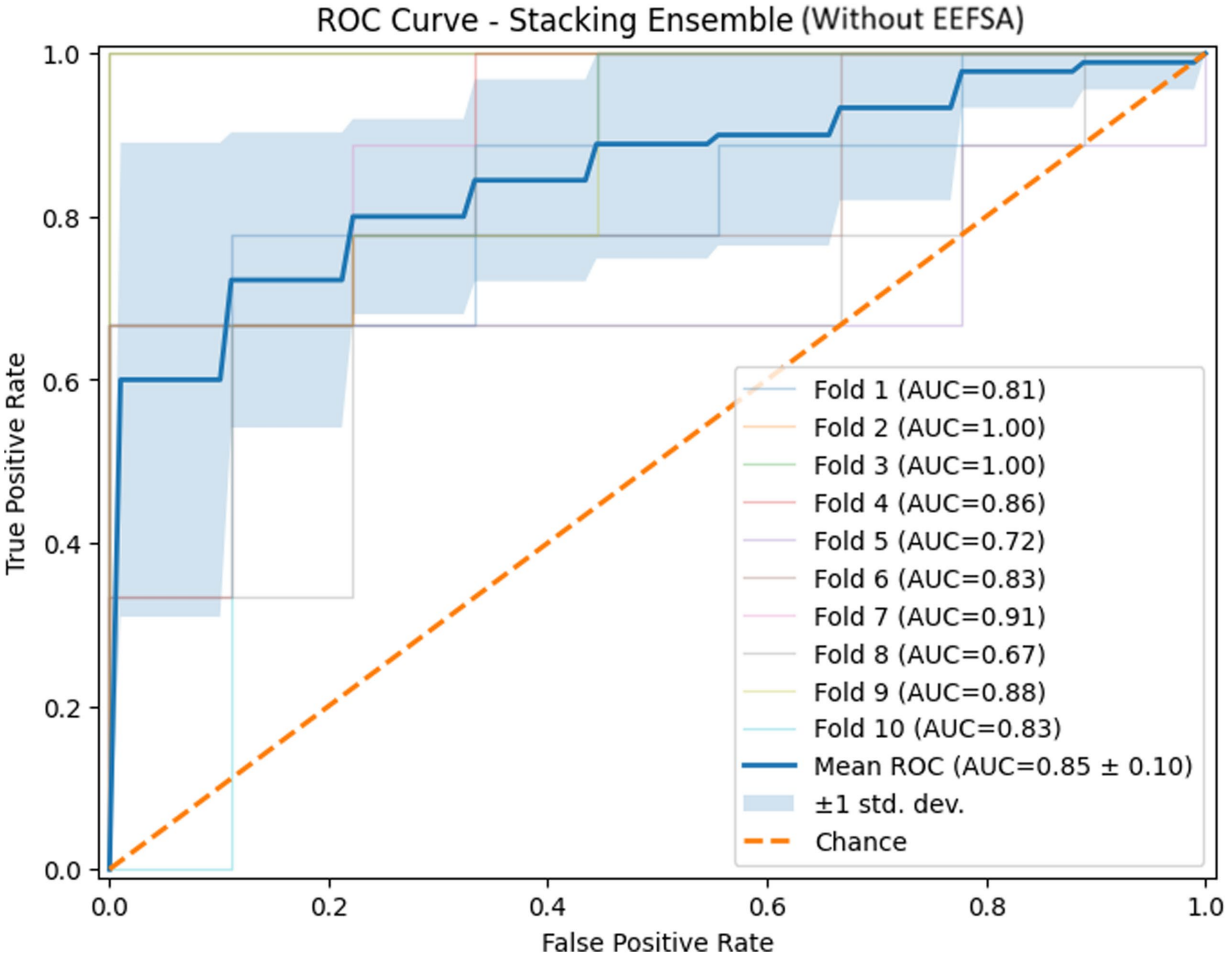
ROC curve of the proposed stacking classifier on Dataset-I using 10-fold CV without ensemble feature selection.

**Figure 7:**
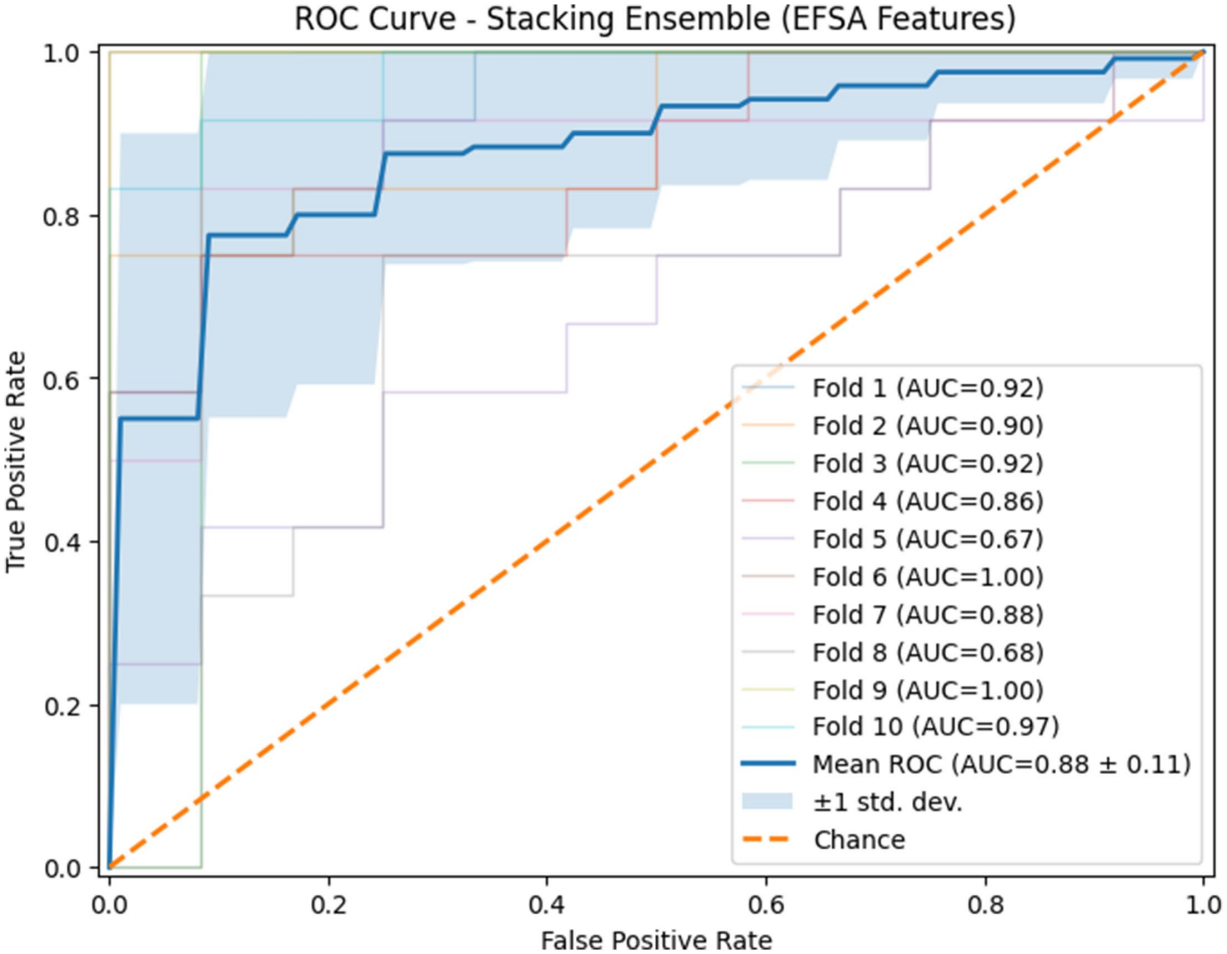
ROC curve of the proposed stacking classifier on Dataset-I using 10-fold CV with ensemble feature selection.

**Figure 8:**
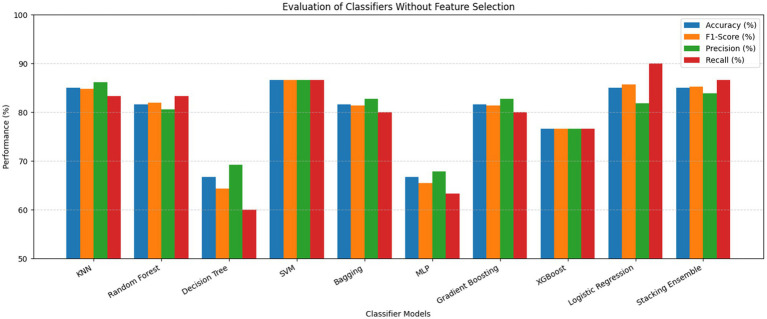
Comparison of performances without ensemble feature selection for Dataset-I.

**Figure 9:**
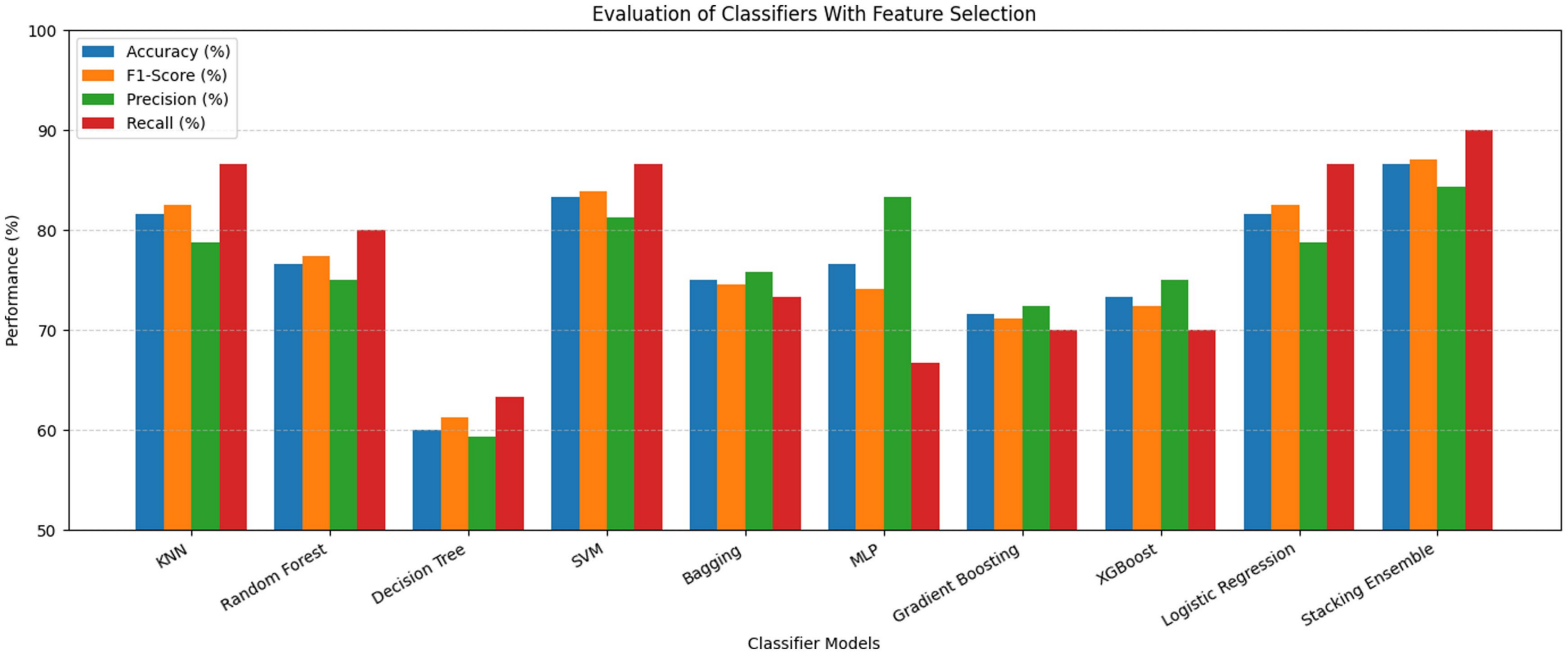
Comparison of performances with ensemble feature selection for Dataset-I.

The ROC curves of the stacking ensemble under 10-fold cross-validation are shown in Figures 6, 7. Without EEFSA, the stacking ensemble achieved a mean ROC-AUC of 0.85 ± 0.10, whereas with EEFSA the mean ROC-AUC increased to 0.88 ± 0.11. This improvement indicates enhanced discriminative capability and slightly improved robustness of the stacking ensemble when trained on the EEFSA-selected feature subset despite overlap in standard deviation changes. The performance results of the classifiers are shown in Figures 8, 9 without and with EEFSA projecting the accuracy, precision, recall, and F1-score.

From a computational perspective, EEFSA also improved efficiency at the model evaluation stage. With feature selection, the stacking ensemble achieved a reduced per-model training time of 6.38 s, compared to substantially higher training times for complex individual models such as MLP (80.34 s), while maintaining competitive testing time. These results demonstrates that the EEFSA framework improves the classification accuracy and generalization while streamlining the computational efficiency. It is an effective approach for strong and adaptable machine learning workflows in Parkinson’s disease classification.

### 4.2 Experiment—Dataset-II

In Dataset 2, as a filtering technique, Pearson Correlation was first used to filter features that have a correlation coefficient greater than 0.30. This resulted in the retention of 15 features. The wrapper approach was then applied using Recursive Feature Elimination (RFE) with a Random Forest estimator which identified 15 important features using a repetitive elimination process. The embedded approach, LassoCV, was used as the third approach to find the best value for the regularization parameter using an alpha grid ranging from 10^−4^ to 10^1^, with 5-fold cross-validation, a maximum of 4,000 iterations leading to the retention of 15 important features based on their coefficients. These three feature subsets combined were then used to form the EEFSA method where Random Forest feature importance scores using Random Forest as the base estimator with 100 decision trees (*n*_estimators = 100) were used to rank the combined features. The top 40 features were then selected to form the EEFSA subset for the training of the model to enhance efficiency and accuracy. These ensemble-selected features were effectively applied using the proposed stacking ensemble approach achieving higher accuracy scores compared to individual feature selection methods. The EEFSA method that selected 40 features outperformed the individual feature selection methods across all evaluation metrics.

The EEFSA-selected features for Dataset-II capture key acoustic patterns in Parkinson’s disease speech. Several MFCC-based features (e.g., mean_MFCC_0th_coef, mean_MFCC_1st_coef, mean_MFCC_2nd_coef, mean_MFCC_6th_coef) describe spectral shape of speech and are sensitive to articulatory imprecision and reduced speech clarity, which are common in PD. A large proportion of the selected features are derived from TQWT and TKEO-based measures, which describe energy distribution, entropy, and nonlinear oscillatory behavior of the speech signal. They show irregular vocal fold vibration, reduced speech stability, and abnormal energy modulation, which are frequently observed in Parkinsonian speech. The features also include GNE_SNR_SEO, GNE_SNR_TKEO, DFA, and IMF-based features, which describe breathiness, reduced harmonic structure, and long-term signal irregularities, monotone speech, and reduced rhythmic control. The complete list of features selected at each stage of the EEFSA process for Dataset-II is provided in the Supplementary material. By combining, these complementary spectral, temporal, and nonlinear features EEFSA-based stacking ensemble achieves improved accuracy and generalization on Dataset-II.

The classifiers are evaluated using the complete feature set and with the EEFSA-selected subset of features. All classifiers and the proposed stacking ensemble model underwent the hyperparameter tuning using GridSearchCV to have optimal model configurations in the similar working conditions. The final stacking ensemble consisted of the top five classifiers selected based on cross-validation accuracy, namely MLP, Gradient Boosting, XGBoost, Random Forest, and Bagging, as determined during the training phase. Table 3 presents the comparative performance of all the classifiers with the complete features. The results in Table 4 shows the performance of all classifiers with the EEFSA-reduced features. The defined Key Performance Indicators (KPIs) were accuracy, F1, ROC, AUC, precision, recall, and even the time taken to train and test the model.

**Table 3:** Performance comparison of classifiers on Dataset-II without EEFSA feature selection.

Model	Accuracy (%)	F1-score (%)	Precision (%)	Recall (%)	MCC (%)	AUC	CV accuracy (10-fold)	CV AUC (10-fold)	Training time (sec.)	Test time (sec.)
KNN	70.90	78.43	98.04	65.36	47.12	0.814	70.55 ± 7.76	75.45 ± 8.55	9.37	0.0248
Random forest	79.37	86.51	91.91	81.70	44.71	0.902	89.36 ± 6.86	82.69 ± 7.47	607.76	0.0565
Decision tree	84.66	90.10	94.29	86.27	57.39	0.820	73.21 ± 6.86	67.46 ± 8.49	48.03	0.0111
SVM	81.48	87.97	92.75	83.66	49.44	0.850	80.95 ± 7.05	83.95 ± 11.91	110.34	0.0206
Bagging	81.48	87.89	93.38	83.01	50.71	0.876	81.00 ± 10.15	83.98 ± 8.27	1628.05	0.0801
MLP	84.66	89.97	95.59	84.97	59.71	0.852	81.12 ± 7.78	82.29 ± 11.84	458.10	0.0122
Gradient boosting	86.24	91.16	95.04	87.58	61.47	0.899	83.25 ± 9.47	85.69 ± 8.89	3015.37	0.0121
XGBoost	82.01	88.28	93.43	83.66	51.58	0.859	83.78 ± 10.51	86.88 ± 9.63	1119.43	0.2040
Logistic regression	80.42	86.83	95.31	79.74	52.97	0.880	82.72 ± 7.16	86.25 ± 13.04	600.18	0.0075
Stacking ensemble	85.19	90.48	94.33	86.93	58.37	0.896	82.21 ± 9.71	87.96 ± 8.38	764.88	0.2523

**Table 4:** Performance comparison of classifiers on Dataset-II with EEFSA feature selection.

Model	Accuracy (%)	F1-score (%)	Precision (%)	Recall (%)	MCC (%)	AUC	CV accuracy (10-fold)	CV AUC (10-fold)	Training time (sec.)	Test time (sec.)
KNN	74.69	82.11	95.56	71.90	46.57	0.835	76.48 ± 8.90	81.85 ± 10.07	4.41	0.0864
Random forest	87.30	91.79	96.04	87.58	65.66	0.898	85.37 ± 7.60	87.96 ± 7.61	485.40	0.0555
Decision tree	75.66	83.27	94.21	74.51	45.05	0.775	79.40 ± 6.26	77.64 ± 7.12	4.83	0.0054
SVM	83.60	89.27	94.85	84.31	56.76	0.889	82.05 ± 6.07	86.82 ± 10.88	31.87	0.0134
Bagging	84.13	89.58	95.56	84.31	58.80	0.881	82.42 ± 7.95	87.90 ± 10.70	173.76	0.0425
MLP	86.24	90.97	97.04	85.62	64.77	0.920	84.51 ± 9.07	85.87 ± 12.34	299.35	0.0063
Gradient boosting	86.24	91.03	96.36	86.27	63.65	0.899	84.35 ± 9.75	83.44 ± 7.39	306.72	0.0058
XGBoost	80.42	87.01	93.94	81.04	50.33	0.877	82.96 ± 7.77	87.81 ± 7.80	92.10	0.0108
Logistic regression	70.37	78.13	97.09	65.40	44.97	0.887	76.54 ± 6.90	85.14 ± 11.18	21.93	0.0039
Stacking ensemble	89.95	93.56	97.18	90.92	71.85	0.900	84.14 ± 8.09	89.52 ± 8.43	69.31	0.1827

The classifiers were evaluated both with the complete feature set and with the EEFSA-selected feature subset. Without feature selection, Gradient Boosting achieved the highest test accuracy of 86.24%, followed closely by the stacking ensemble with an accuracy of 85.19% (Table 3).

Although the stacking ensemble involves multiple base learners during model selection, the reported computational time reflects the final model training and inference cost under identical evaluation conditions. Under this setting, Gradient Boosting required a substantially higher per-model training time (3015.37 s), whereas the stacking ensemble required 764.88 s, while maintaining competitive discriminative performance (ROC-AUC = 0.896).

After applying EEFSA, a clear improvement was observed across nearly all classifiers (Table 4). The stacking ensemble achieved the best overall performance, with a test accuracy of 89.95%, F1-score of 93.56%, precision of 97.18%, recall of 90.92%, and the highest MCC of 71.85%. In addition, EEFSA significantly reduced the per-model training cost of the stacking ensemble to 69.31 s, while preserving a low testing time of 0.1827 s.

These results indicate that EEFSA enhances not only predictive performance but also computational efficiency at the model evaluation stage, making the proposed stacking framework suitable for practical deployment on large-scale acoustic datasets.

**Figure 10:**
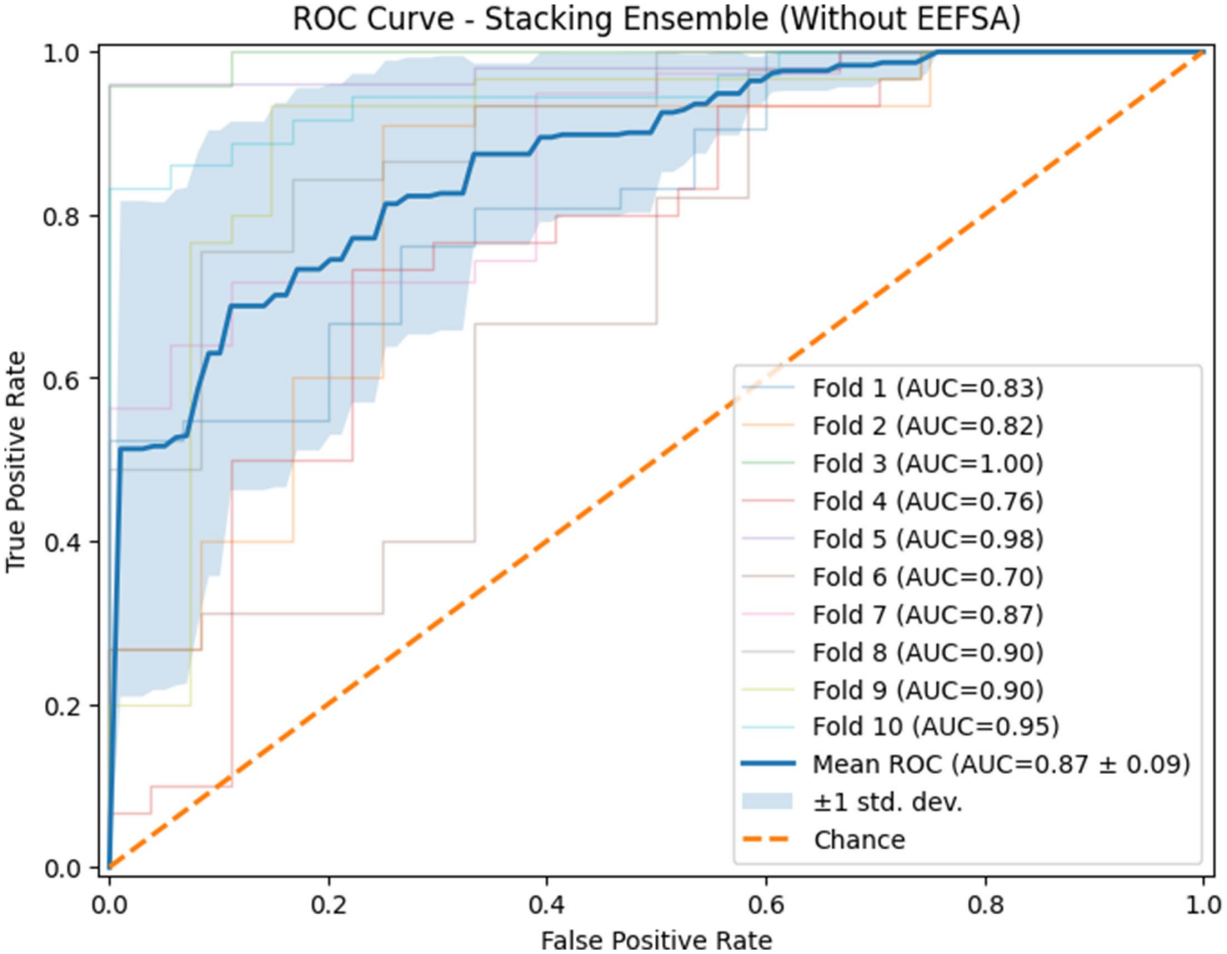
ROC curve of the proposed stacking classifier on Dataset-II using 10-fold CV without ensemble feature selection.

**Figure 11:**
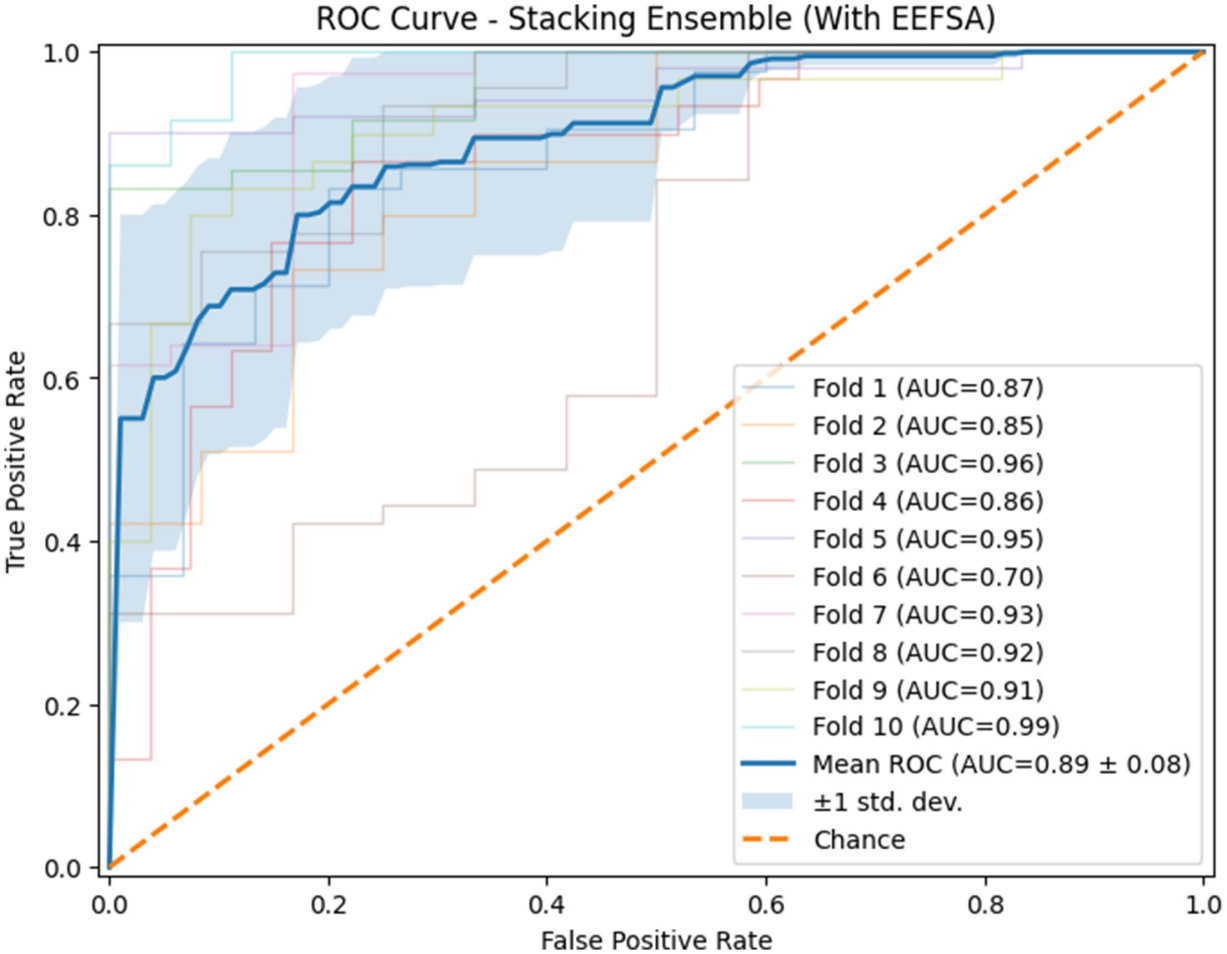
ROC curve of the proposed stacking classifier on Dataset-II using 10-fold CV with ensemble feature selection.

**Figure 12:**
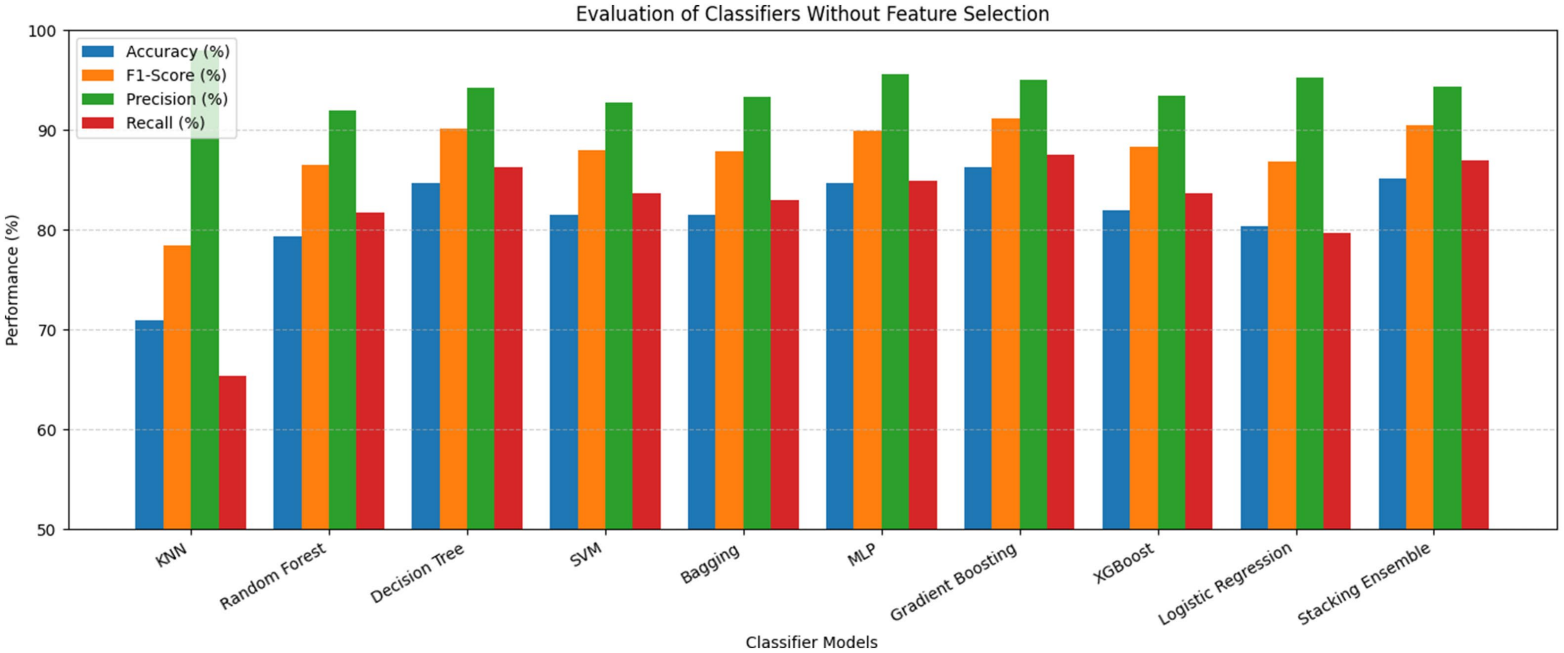
Performance comparison without ensemble feature selection for Dataset-II.

**Figure 13:**
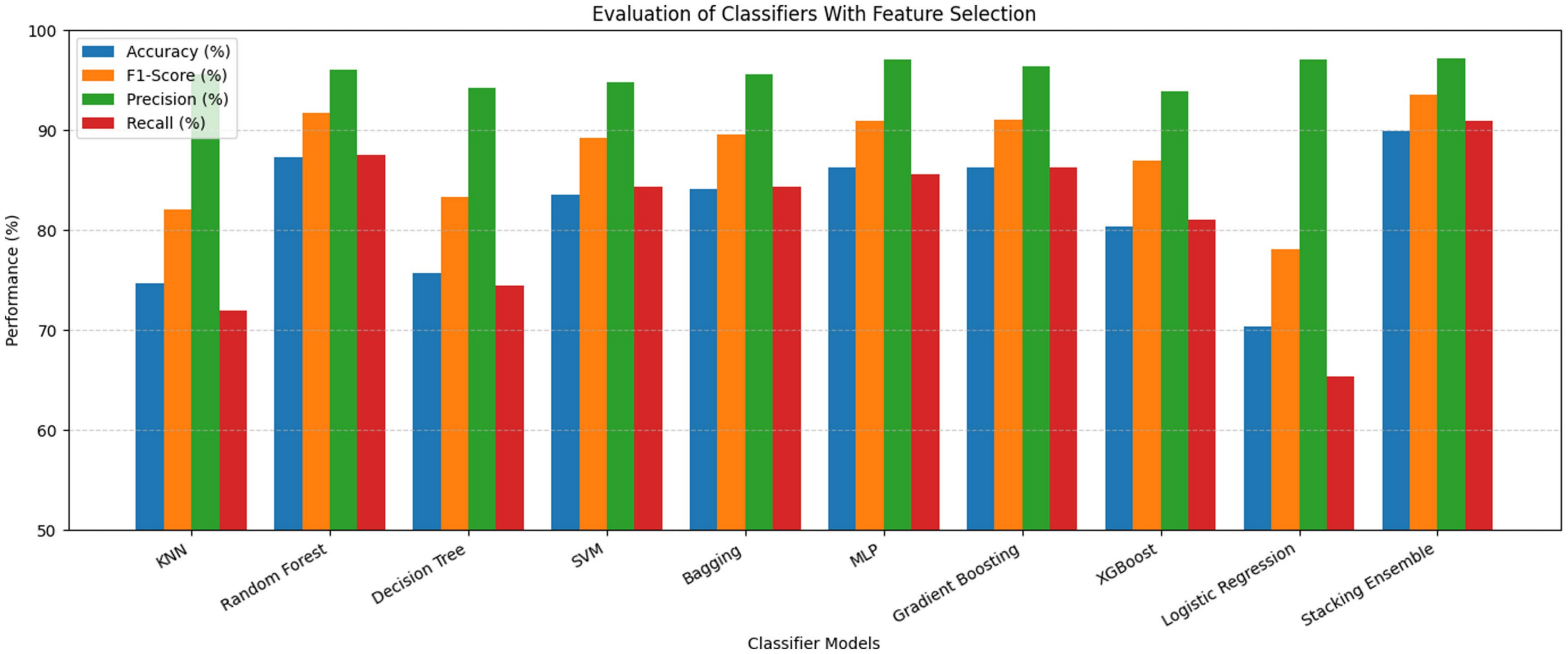
Performance comparison with ensemble feature selection for Dataset-II.

Using EEFSA methods, most classifiers showed increased performance across all evaluation metrics. Figures 10, 11 illustrates the ROC curves of the stacking ensemble classifier under 10-fold cross-validation both without and with EEFSA. Without EEFSA, the model achieved a mean ROC- AUC of 0.87, while with EEFSA, the mean ROC-AUC improved to 0.89 figure but with lower variance, illustrating better model generalization and trustworthiness of the model. The performance of different classifiers was evaluated and compared on Accuracy, Precision, Recall and F1 Score (Figures 12, 13). EEFSA feature selection enhanced performance across all models but proved most effective on Ensemble Stacking and Gradient Boosting classifiers.

The evaluation results indicate that applying EEFSA leads to consistent improvements in test accuracy and stability compared to using the complete feature set. In addition, EEFSA reduces the per-model training and inference cost for most classifiers. The stacking ensemble model, which obtained the highest accuracy at the lowest per-model evaluation time, confirms the dual benefits fetch by EEFSA. The results obtained clearly demonstrate the applicability of the EEFSA framework on Dataset II, which enhanced classification accuracy, reduced overfitting tendencies, and optimized computation resource expenditure.

## 5 Performance comparison

The comparison results presented in Table 5 show that existing methods on Dataset-I achieved accuracies ranging from 75% to 84.17%, with Singh and Tripathi (19) reaching 83.30% using a hybrid feature selection approach. In contrast, the proposed EEFSA-SECM model achieved 86.67%, outperforming prior work and achieves the highest accuracy among classical ML approaches. For Dataset-II presented in Table 5, as in earlier studies reported accuracies between 80% and 91.62%, with Sabeena et al. (14) achieving the highest accuracy of 98.77% using a deep learning-based approach. The proposed EEFSA-SECM attained 89.95%, which is competitive with the best-performing models, while maintaining efficiency by relying on a reduced and more discriminative feature subset. These findings demonstrate that EEFSA-SECM offers a favorable balance between classification accuracy and feature selection efficiency, making it a robust solution for Parkinson’s disease detection.

**Table 5:** Summary table for comparison of suggested feature selection performance to recent studies on the PD dataset.

Dataset	References	FS method	Technology	Accuracy
Dataset-I UCI Parkinson dataset with replicated acoustic features data set	(23)	40PD, 40HC	Bayesian classification (mean)	75%
	(12)	21, RFE	Bagging with recursive feature elimination	82.31%
	(24)	40PD, 40HC	Proposed model MI-AE-GOLGBM	84.17%
	(19)	17 features	Correlation + OLS *p*-value + LASSO	83.30%
	Proposed (EEFSA-SECM)	EEFSA algorithm (selected 20 features)	SECM classifier	86.67%
Dataset-II UCI Parkinson’s disease classification data Set	(18)	All features	Ensemble with voting	81%
			Ensemble with stacking	80%
		mRMR-50 and the TQWT	SVM (RBF kernels)	86%
	(25)	50 features	SHAP-light GBM	91.62%
	(26)		Majority of voting	84.48%
	(14)		(MFCC + wavelet + concat), FCBi-LSTM classifier (%)	98.77%
	(27)	All features	Feature reduction principal component analysis (PCA)	91%
	(19)	45 features	Correlation + OLS *p*-value + LASSO	90.20%
	Proposed (EEFSA-SECM)	EEFSA algorithm (selected 40 features)	SECM classifier	89.95%

## 6 Conclusion

This framework is built combining Enhanced Ensemble Feature Selection Algorithm (EEFSA) with Stacking Ensemble Classifier model (SECM) aimed to improve the machine learning models built on biomedical datasets. EEFSA is based on three feature selection strategies namely filter, wrapper, and embedded methods which use Pearson Correlation, Recursive Feature Elimination, and LassoCV, thus assuming an optimal feature space by enforcing dimensionality reduction is performed without losing the discriminative power. The determined feature set was processed by feature selection in which the selected features were ranked and aggregated to form a strong feature subset that improved the predictive accuracy while reducing the time in computation.

Experimental results on Dataset-I and Dataset-II demonstrated that the proposed stacking ensemble consistently outperformed traditional classifiers across multiple evaluation metrics. Notably, the model achieved competitive accuracy and F1-scores while significantly reducing training and testing time, especially when EEFSA was applied. The ROC curves as well as the cross-validation scores also corroborate the trustworthiness and the generalizability of the model.

As a result, the combination of EEFSA along with a stacking ensemble classifier model (SECM) provides a powerful, computationally efficient, interpretable, and rapid solution to high dimensional classification problems, especially in the biomedical field. This approach can be extended to other classification problems in healthcare, real-time diagnostic systems, and multimodal datasets integrating various data modalities.

## Data Availability

Publicly available datasets were analysed in this study. This data can be found here: https://archive.ics.uci.edu/dataset/489/parkinson+dataset+with+replicated+acoustic+features, https://archive.ics.uci.edu/dataset/470/parkinson+s+disease+classification.
