# Supplementary material for: EEFSA-SECM: an enhanced ensemble feature selection and stacking ensemble classifier to detect Parkinson’s disease
Source: Front Neurol. 2026 Mar 2;17:1717252. doi: 10.3389/fneur.2026.1717252 (PMC12989349; doi:10.3389/fneur.2026.1717252)
Supplement: Supplementary file 1 [file Table_1.pdf]

Final Hyperparameters selected by Grid SearchCV for Dataset-I and Dataset-II

C=0.1, kernel, gamma=scale(svm), n-neighbors,weights(knn), max depth, minsplit(random forest), c, l1ratio(logistic regression), hiddenlayers,alpha(MLP), n-estimators,max-samples, max depth,learning rate(bagging,boosting), maxdepth,min split(decision tree)

Features Selected by EEFSA Methods using Dataset-I

Filter (Pearson) -MFCC10; MFCC5; MFCC12; Delta11; MFCC3; Delta3; HNR35

Wrapper (RFE) - HNR35; HNR38; MFCC3; MFCC8; MFCC10; MFCC12; Delta2; Delta3; Delta11; Delta12

Embedded (LassoCV) - Jitter_PPQ; Shim_APQ5; HNR35; MFCC2; RPDE; gender; DFA; PPE; GNE; MFCC1

Union (EFSA) - DFA; Delta11; Delta12; Delta2; Delta3; GNE; gender; HNR35; HNR38; Jitter_PPQ; MFCC1; MFCC10; MFCC12; MFCC3; MFCC2; MFCC5; MFCC8; PPE; RPDE; Shim_APQ5

Final Selected (EFSA) - PPE; MFCC3; MFCC2; GNE; HNR35; DFA; MFCC10; Delta3; Delta11; HNR38; Shim_APQ5; Delta2; gender; MFCC1; Delta12; Jitter_PPQ; MFCC5; RPDE; MFCC12; MFCC8

Features Selected by EEFSA Methods using Dataset-II

Filter (Pearson) - mean_MFCC_2nd_coef; tqwt_minValue_dec_12; tqwt_entropy_log_dec_12; tqwt_stdValue_dec_12; tqwt_maxValue_dec_12; std_8th_delta_delta; std_6th_delta_delta; std_9th_delta_delta; std_7th_delta_delta; tqwt_minValue_dec_13; tqwt_maxValue_dec_13; tqwt_entropy_shannon_dec_15; tqwt_stdValue_dec_11; tqwt_energy_dec_12 std_8th_delta

Wrapper (RFE) - std_delta_log_energy; std_delta_delta_log_energy; std_6th_delta_delta;std_9th_delta_delta;app_LT_entropy_shannon_8_coef; tqwt_energy_dec_26;tqwt_energy_dec_27;tqwt_entropy_log_dec_35; tqwt_TKEO_mean_dec_12;tqwt_TKEO_std_dec_6;tqwt_TKEO_std_dec_12;

tqwt_stdValue_dec_12;tqwt_stdValue_dec_18;tqwt_maxValue_dec_13; tqwt_kurtosisValue_dec_20

Embedded (LassoCV) - DFA; stdDevPeriodPulses; meanIntensity; f1; f2; f3; b4; GNE_SNR_TKEO; GNE_SNR_SEO; IMF_NSR_TKEO; IMF_NSR_entropy; mean_MFCC_0th_coef; mean_MFCC_1st_coef; mean_MFCC_6th_coef

Union(EFS) – DFA; GNE_SNR_SEO; GNE_SNR_TKEO; IMF_NSR_TKEO; IMF_NSR_entropy; app_LT_entropy_shannon_8_coef; b4; f1; f2; f3; meanIntensity;mean_MFCC_0th_coef;mean_MFCC_1st_coef; mean_MFCC_2nd_coef; mean_MFCC_6th_coef; stdDevPeriodPulses; std_6th_delta_delta; std_7th_delta_delta; std_8th_delta; std_8th_delta_delta; std_9th_delta_delta;std_delta_delta_log_energy;std_delta_log_energy; tqwt_TKEO_mean_dec_12; tqwt_TKEO_std_dec_12; tqwt_TKEO_std_dec_6; tqwt_energy_dec_12;tqwt_energy_dec_26;tqwt_energy_dec_27; tqwt_entropy_log_dec_12;tqwt_entropy_log_dec_35; tqwt_entropy_shannon_dec_15;tqwt_kurtosisValue_dec_20; tqwt_maxValue_dec_12;tqwt_maxValue_dec_13;tqwt_minValue_dec_12; tqwt_minValue_dec_13;tqwt_stdValue_dec_11;tqwt_stdValue_dec_12; tqwt_stdValue_dec_18

Final Selected (EFSA) - tqwt_minValue_dec_12; std_6th_delta_delta; tqwt_maxValue_dec_13; DFA; b4; std_delta_log_energy; tqwt_stdValue_dec_12; mean_MFCC_0th_coef;GNE_SNR_TKEO;tqwt_energy_dec_26; tqwt_TKEO_std_dec_6;std_7th_delta_delta;tqwt_kurtosisValue_dec_20; stdDevPeriodPulses;GNE_SNR_SEO;tqwt_energy_dec_27; app_LT_entropy_shannon_8_coef; std_9th_delta_delta; f3; f2; mean_MFCC_1st_coef; std_8th_delta_delta; tqwt_TKEO_std_dec_12; f1;std_8th_delta;meanIntensity;tqwt_stdValue_dec_18; tqwt_entropy_log_dec_35; mean_MFCC_2nd_coef; tqwt_stdValue_dec_11; tqwt_entropy_shannon_dec_15; tqwt_minValue_dec_13; mean_MFCC_6th_coef; tqwt_TKEO_mean_dec_12; tqwt_maxValue_dec_12; tqwt_energy_dec_12; IMF_NSR_TKEO;tqwt_entropy_log_dec_12;std_delta_delta_log_energy; IMF_NSR_entropy
